# A Concise Analysis of Professors Hufeland and Kant's Treatise on the Art of Prolonging Life

**Published:** 1799-07

**Authors:** 


### *A conciſe Analyſis of Profeſſors* HUFELAND *and* KANT'S *Treatiſe on the Art of prolonging Life.*

[*Concluded from our third Number*, pp. 259—263.]

ADVANCED age, though it be the natural reſult of life, and the firſt ſtep towards death, may nevertheleſs of itſelf become the means of prolonging life. It is true, it does not increaſe the principle of vital ſpirits, but it retards their conſumption; and it muſt be allowed, that man would ſooner terminate terminate the laſt period of his life, at the time when he ſuffers a diminution of his natural powers, if he did not, at the ſame time, grow old. It now remains to explain this aſſertion, which is apparently paradoxical.

In the decline of life, man is leaſt provided with vital ſpirits, and with the faculty of reſtoring thoſe which he daily loſes; if, in the mean time, he continue to live in the ſame active ſtate, and poſſeſs the ſame vivacity as formerly, the ſpirits will be the more readily exhauſted, and he will the ſooner terminate his life; but the character of the preſent age diminiſhes the effect of natural irritation, and of the ſenſitive faculty. Interior as well as exterior objects do not ſtrike him ſo forcibly; conſequently, he exhauſts a leſs quantity of power and energy, and therefore advances ſtill farther with that portion of power which remains; and if he lives temperately, his life will be prolonged. It is to this reduced degree of irritation, that the preſent age owes the faint effect of dangerous impreſſions, and the cauſes of diſeaſe; ſuch as paſſions, violent exertions, &c. It maintains a greater degree of harmony and calmneſs in the interior economy, and by that means fortifies the body againſt various accidents. On this account alſo it is remarked, that old men are leſs ſubject to epidemic attacks than young perſons. To this may be added the manner of living, which inconteſtibly contributes, in a great degree, to the prolongation of life. The operations of the animal economy, which have continued during ſo long a time, in the ſame order and manner, become at length ſo habitual, that they are carried on, as it were, by habit alone, when other cauſes ceaſe to exert their influence. It is often aſtoniſhing, to ſee how individuals of a very advanced age, continue to ſupport themſelves during a certain period, in ſuch a manner, that the whole ſyſtem is ſupported in a progreſſive regular order. When the moral powers of man are almoſt completely annihilated, his phyſical faculties continue to vegetate ſome time longer; the latter of which, doubtleſs require leſs of vital ſupply than if he exiſted in an integral ſtate. It is alſo this ſtate of life which makes a man wiſh to live longer as he increaſes in age. We may venture to add, that this deſire becoming every day more ardent, and conſequently cheriſhing hope, ſupplies perhaps ſtill more the loſs of vital ſpirits, which, as Hufeland has ſomewhere remarked, increaſe with hope.

The ſyſtem of regimen to be followed is, to diminiſh exſiccation, and to ſoften the ſtiffneſs of the fibres, the contractility of which is liable to be totally ſuſpended. It is neceſſery to facilitate the reſtoration of loſſes and digeſtion as much as poſſible. A greater degree of irritation ſhould be given to the body, ſince the natural irritability is conſiderably diminiſhed. The ſecretion of the putrid miaſmata ought to be promoted, which in old age, age, is fo imperfectly formed, and which attracts that impurity of humours that contribute to a fpeedy diffolution. From thefe principles are derived the following rules:

1. In old age the natural heat fails; it is confequently neceffary to fupport it as much as poffible, by means of external objects, warm clothes, warm apartments, warm beds, nourifhing food, and alfo, if poffible, a temperate climate.

2. The food ought to be fuch as is eafy of digeftion, rather liquid than folid, of a concentrated fubftance, and more ftimulating than was formerly conducive to the welfare of the individual. Hence jelly-broths, feafoned with fpices, are of great benefit to old perfons: give them roaft meat, well done, beans, peas, and other nourifhing pulfe, ftrong beer, and above all, plenty of good old wine. which does not contain acid, earthy particles, or vifcidity, fuch as old Spanifh wine, Tokay, Cyprus, and Cape wine. Such wines are to them one of the greateft refources of life; they do not overheat, but nourifh and ftrengthen the aged; in fact they are the balm, the real milk of old age.

3. Tepid baths are of great utility: they are among the beft means of augmenting the natural heat, of promoting the fecretions, particularly cutaneous perfpiration, and of ultimately diminifhing the exficcation and rigidity of the fibres and membranes. They anfwer almoft all the neceffities of this period of life.

4. All violent evacuations fhould be carefully avoided, as venefection, (unlefs in particular, urgent cafes) violent purgatives, as well as fenfible perfpiration, the fexual intercourfe, &c.

5. As foon as a perfon attains an advanced age, he ought to accuftom himfelf in a great degree to arrange all his functions of life, according to a certain order; he ought to reft, take exercife, eat, and drink at regular periods. This order, this mechanical habit of living, contributes in a remarkable degree to prolong life.

6. The body requires exercife, but great care fhould be taken, not to make ufe of any that may affect or exhauft it; a paffive fpecies of exercife fhould be preferred, fuch as riding in carriages, repeated friction with odoriferous and tonic ointments, which diminifh the ftiffnefs of the fibres, and keep the fkin in a healthy ftate. But above all things, care fhould be taken to avoid fudden alarms or fhocks; fuch are commonly the forerunners of death,

7. A

7. A cheerful temper, and agreeable amuſements, produce a wonderful effect on the human body, provided they do not partake of the ſtronger paſſions, which, in an advanced age, may produce effects inſtantly fatal. What are moſt beneficial, are, livelineſs and contentment, the effects of domeſtic happineſs, which are enjoyed by reflecting on the paſt; by perceiving that we have not lived in vain, and even looking towards death with ſatisfaction.

The fooliſh games of childhood, and the ſociety of children, are moſt agreeable to old people. Their infantine queſtions, their innocent joy, ſeem to have a renovating effect upon age. Hope and future proſpects are expedients particularly uſeful; new plans, freſh undertakings (provided they contain nothing dangerous or embarraſſing), in ſhort, every thing which tends to increaſe the expectation of a ſtill further prolongation of life, all contribute to extend its phyſical limits. We find alſo, that in old perſons, this is an inſtinctive idea; they begin to build, to arrange their gardens, and appear to take a ſingular delight in this faint illuſion, from which it may be inferred they wiſh to inſure their lives.

